# Extreme seasonal water-level changes and hydraulic modeling of deep, high-altitude, glacial-carved, Himalayan lakes

**DOI:** 10.1038/s41598-023-37667-z

**Published:** 2023-07-20

**Authors:** R. Camassa, E. F. Eidam, L. G. Leve, R. M. McLaughlin, H. E. Seim, S. Sharma

**Affiliations:** 1 Department of Mathematics, University of North Carolina Chapel Hill, Chapel Hill, USA; 2 College of Earth, Ocean, and Atmospheric Sciences, Oregon State University, Corvallis, USA; 3 Department of Religious Studies, University of North Carolina Chapel Hill, Chapel Hill, USA; 4 Department of Earth, Marine, and Environmental Sciences, University of North Carolina Chapel Hill, Chapel Hill, USA; 5 Department of Environmental Science and Engineering, Kathmandu University, Dhulikhel, Nepal

**Keywords:** Climate sciences, Limnology, Mathematics and computing, Applied mathematics

## Abstract

Himalayan lakes represent critical water resources, culturally important waterbodies, and potential hazards. Some of these lakes experience dramatic water-level changes, responding to seasonal monsoon rains and post-monsoonal draining. To address the paucity of direct observations of hydrology in retreating mountain glacial systems, we describe a field program in a series of high altitude lakes in Sagarmatha National Park, adjacent to Ngozumba, the largest glacier in Nepal. In situ observations find extreme (>12 m) seasonal water-level changes in a 60-m deep lateral-moraine-dammed lake (lacking surface outflow), during a 16-month period, equivalent to a 5 $\times 10^6$ m$^3$ volume change annually. The water column thermal structure was also monitored over the same period. A hydraulic model is constructed, validated against observed water levels, and used to estimate hydraulic conductivities of the moraine soils damming the lake and improves our understanding of this complex hydrological system. Our findings indicate that lake level compared to the damming glacier surface height is the key criterion for large lake fluctuations, while lakes lying below the glacier surface, regulated by surface outflow, possess only minor seasonal water-level fluctuations. Thus, lakes adjacent to glaciers may exhibit very different filling/draining dynamics based on presence/absence of surface outflows and elevation relative to retreating glaciers, and consequently may have very different fates in the next few decades as the climate warms.

## Introduction

The Himalayas presently possess a vast amount of water stored in ice. This region, often known as the third pole, provides crucial water sources for Asia, a region populated by over 4 billion human beings. As the planet warms, mountain glaciers worldwide are generally retreating^1^, a process which can lead to the formation of moraine-dammed and ice-marginal lakes in periglacial and transitional alpine environments^2–5^, and the potential energy stored in this ice ultimately will be lost to the oceans as a fundamental water resource will disappear. These newly forming alpine lakes can provide new habitats and important water sources for nearby communities, pose hazards in the form of natural dam failures^6,7^, and further provide important benchmark indicators for assessing the future impacts of climate change which may occur elsewhere, such as in Earth’s polar regions. Since the Himalayas are warming at among the most rapid rates anywhere on the planet^8^, the third polar region is an important area to study as it provides potential insight into future climate change at higher latitudes. The rapid evolution and seasonal to interannual dynamics of these lakes are not well studied, but increasingly important to understand. In some environments, such lakes have been a target of flood concern, especially in cases where the natural moraine dam may fail and/or the lake may overtop its basin; in other environments, there is concern that water levels may decrease until the lakes are no longer viable water sources^9–15^.

**Figure 1:**
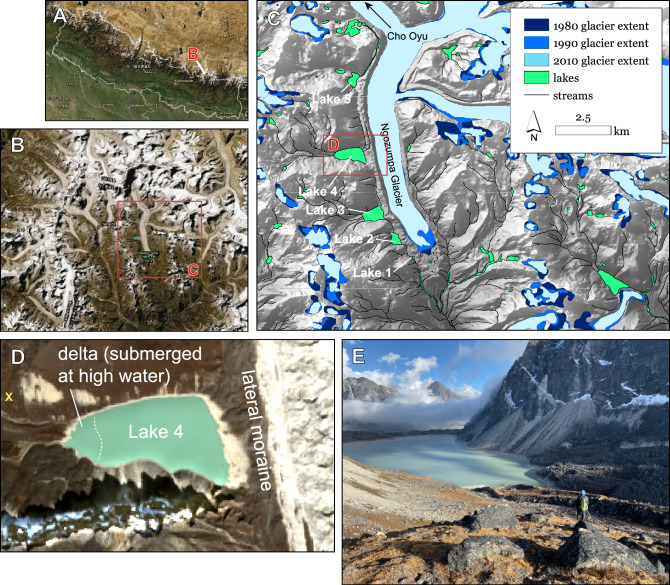
Vicinity map. (**A**) Nepal. (**B**) Ngozumpa Glacier within Sagarmatha National Park. (**C**) Multi-decadal changes in extents of Ngozumpa Glacier and nearby hanging glaciers, with locations of lakes 1–5 noted. Lake 4 was the primary location of this study. Additional measurements were collected from or near lakes 1, 2, 3, and 5. (**D**) Lake 4, the primary study focus, with yellow “X” marking location where (**E**) photo was taken. (**E**) Lake 4 viewed from the northwest corner looking southeast. Satellite images were obtained from USGS Earth Explorer (https://earthexplorer.usgs.gov/) (**A,B**) and Sentinel Hub (**C**).

Here, we present results and analysis of a field campaign focused on the Gokyo Lakes, a high-altitude lake system within Sagarmatha National Park, Nepal. This lake system, adjacent to the Ngozumpa Glacier (the longest glacier in Nepal, over 20 km long), is located west of Mt. Everest, and consists of a series of six lakes (Fig. 1). The three largest lakes, ranging in depth from 30 to 60 m^16^, have formed in valleys carved by tributary glaciers and are dammed by the large western lateral moraine of Ngozumpa Glacier. This is a debris-covered glacier whose sediment sources include bedrock scour and landslides^17^.

The Gokyo lakes are generally referred to by numbers (from Lake 1 in the south to Lake 6 in the north). Lakes 1, 2 (Taujung Tso), and 3 (Gokyo Tso) are all connected by surface streams to the headwaters of the Dudh Koshi, a tributary which eventually feeds the Ganges-Brahmaputra River outside of Nepal. These lower lakes all have discharge through surface streams. In contrast, Lake 4 (Thonak Tso) and Lake 5 (Ngozumba Tso) lack surface outflows.

Because of interest in the hydrology of these lakes, the annual water budget is clearly relevant. The Gokyo region lies in the subtropics and has a climate impacted by the Southwest Asian monsoon. Precipitation peaks during July and August. Air temperatures are typically above freezing between May and September. Based on meteorological data from the Pyramid Observatory in the adjacent Khumbu Valley (nearly 12 km due east of Gokyo), total annual precipitation is approximately 0.45 m, and more than three-quarters of this falls between June and September^18^. Together with glacial melt, these water sources feed this lake system, and provide necessary boundary conditions for predicting the system’s evolution, but the hydrology of these lakes is poorly documented and not understood. In this study, we report a 16-month time series of Lake 4 water levels and thermal structure to document annual lake dynamics. This time series shows dramatic seasonal lake level variations which require explanation. To this end, a simplified hydraulic model of lake filling and draining is derived, analyzed, and compared to observational data. The model allows us to identify and validate which terms of the water budget are most important to the hydrology of these lakes, and in particular to help estimate unknown hydraulic conductivity of the damming moraine terrain as well as to rule out secondary effects such as evaporation. Our measured thermal structure evolution allows us to utilize a recent classification scheme for ice-covered lakes^19^ to characterize the Gokyo lakes as cryostratified. We then discuss the implications of these findings.

## Methods and materials

We next discuss our observations and available precipitation data sets which we in turn use to develop a forecast model for better understanding the dynamics and regulatory mechanisms of this lake system. As discussed in the Supplementary Material, water level and temperature time series from Lake 4 were recorded during a 16 month period, and precipitation measurements from two sources were utilized in our hydraulic modeling. For our water level and temperature time series, we utilized a string of 10 Onset HOBO water temperature Pro v2 (U22-001) data loggers, bounded top and bottom by Onset HOBO water level data loggers (U20L-002 above and U20-001-03 below), spaced approximately 4 m apart. The bathymetry of Lake 4 was established by combining soundings measured with the Deeper Pro echosounder with discrete depth measurements from the RBR profiler and satellite-derived topography. Please see Supplementary Material Fig. S3 for precise locations of where data were collected on Lake 4.

We utilized two distinct precipitation data sets in our modeling effort below. First, observations were collected at the Pyramid International Laboratory/Observatory (27$^\circ$57$^{\prime }$ 33$^{\prime \prime }$ N, 86$^\circ$48$^\prime$ 47$^{\prime \prime }$ E^18^) located in the Khumbu valley roughly 12 km east of Lake 4. Second, reanalysis data from ERA5-land (https://www.ecmwf.int/en/era5-land) were downloaded for a grid cell centered over Lake 4 at hourly intervals for the a longer time period and were used to generate a model forecast of varying lake depths. For further details, please see the Supplementary Material.

## Observed results and hydraulic modeling validation

**Figure 2:**
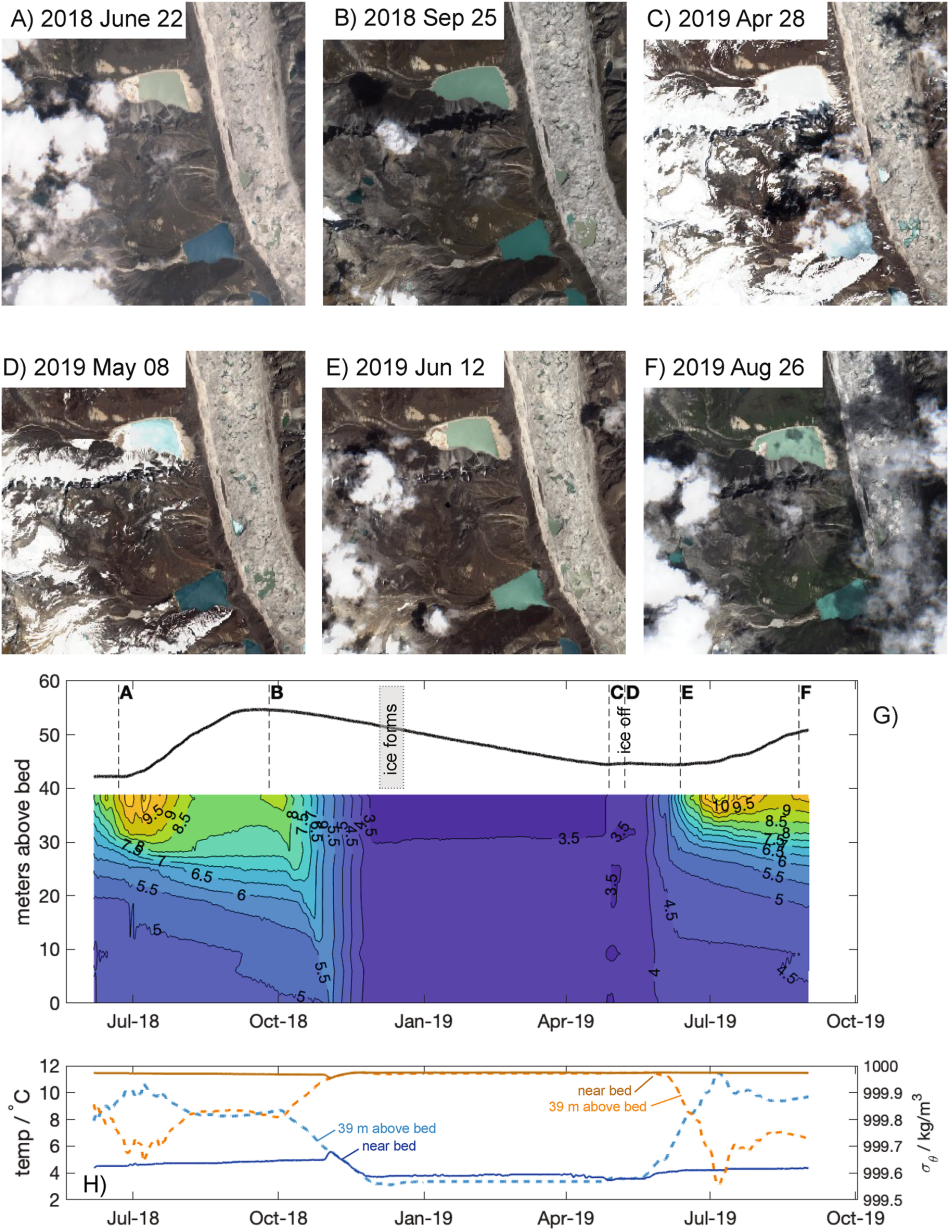
Changes in lake levels and ice cover. (**A–F**) Satellite images from Sentinel Hub of lakes 3 (bottom) and 4 (top) corresponding to times in (**G**). (**G**) Measured water-level record and thermal structure from lake mooring. Ice formed after the water column became well-mixed (late Nov 2018). The lake level rose slightly while ice was thinning. The lake level was nearly constant between mid-May and mid-June, while stratification was developing. The lake level began to rise in late June. First time tick-mark is July 1, 2018. (**H**) Time series of temperature (blue) and potential density (orange) from the lake bottom (solid) and 39 m above lake bottom (dashed).

Between June and September 2018, the Lake 4 water depth increased from 42.2 to 54.7 m, an increase of 12.5 m, in response to the summer monsoon (Fig. 2). During this time the lake was stably stratified, with water temperatures ranging from less than 6 $^{\circ }$C in the lower 20 m, increasing to more than 11 $^{\circ }$C near the surface. Based on our hypsometry, this represented a change in lake volume from 8.76 $\times$ 10$^{6}$ to 14.4 $\times$ 10$^{6}$ m$^3$ (approximately $5.6 \times 10^6$ m$^3$). When extrapolated to a total catchment area of 15.9 km$^2$ (estimated from Google Earth images and topography), this represents a net seasonal precipitation amount from all sources of 0.35 m (or 0.007 m per day) assuming no evaporation or infiltration.

The lake depth at the mooring location (see Supplementary Fig. S3 for location and Lake 4 bathymetry) remained near its maximum ($\simeq 54.7$ m) from 3 Sep to 12 Oct 2018. Between 12 Oct 2018 and 25 April 2019 (195 days), the lake depth decreased from 54.2 m to 44.5 m (a change of 9.7 m), representing an average decrease of about 0.0497 m/day. Volumetrically, this represented a decrease of 23, 795 m$^3$/day. Lake temperatures began to cool in early October, as lake depth began decreasing (Fig. 2). A surface mixed layer developed that deepened to the full depth of the lake by early November; lake temperatures at the onset of well-mixed conditions were $5.5^{\circ }$ C. By late November lake temperatures cooled to below $4^{\circ }$C. Satellite imagery indicates, see Fig. 2 panels A–F, the lake iced-over in mid-December, at which time the lake was weakly thermally stratified, a roughly $0.5^{\circ }$C change vertically, with cooler waters near the surface because the fluid was all below the temperature of maximum density for fresh water (roughly $4^{\circ }$C). Lake temperature remained unchanged until early April when near-surface waters began to warm, and in late April the lake overturned producing well-mixed conditions. Lake depth stopped decreasing in late April, coincident with overturning. Lake depth remained approximately constant until mid-June, when it began to rise again. Thermal conditions were well-mixed from late April until mid-May, after which time surface waters warmed rapidly, re-establishing stable stratification. In early July lake levels began rising and continued to do so until the measurements end in early September. For temperature and turbidity profiles collected in mid-October in lakes 2, 4 and 5, please see the Supplementary materials document.

Given the morphology of the terrain where the land-locked Lake 4 is located, it is reasonable to assume that most of the underground outflow from the lake is directed towards the Ngozumba glacier, and is concentrated along the east side of the lake, where the glacier’s moraine effectively acts as a dam wall. From our echosounder data, we construct the curve of this wall’s length vs. depth $L_w(z)$, as well as the hypsometric curve *A*(*z*) for the lake, where the origin of the vertical axis *z* is set at the maximum depth point of the lake, see the Supplementary material for further details. Assuming Darcy’s law, we derive (see the Supplementary Materials for details) the following integral-differential equation for the Lake 4 depth evolution, *h*(*t*)

1 $$\begin{aligned} {d h\over dt}={1\over A\big (h(t)\big )}\left( Q_{ in}(t) -K\int _0^{h(t)} A_w(z)dz \right) . \end{aligned}$$

Here, *A*(*h*(*t*)) is total lake area at level $z=h(t)$, $Q_{ in}(t)$ is the total volumetric influx, $A_w(z)$ is the area of the east lateral moraine wall, defined by the integral of wall’s length, $L_w(z)$, and the constant, *K*, is proportional to the hydraulic conductivity, $K_h$. Notice in Fig. 2, in June of 2018 the depth is roughly constant, indicating a balance of the inflow with the porous media ground flux outflow. Also notice the strongly linear decay rate between October 2018 through April 2019. These two pieces of the data can be used to estimate the hydraulic conductivity, $K_h=37.42$ m/day, which is in line with field-experimental measurements of conductivity for glacial tills (e.g., Eklutna Valley, southcentral Alaska^20^, Lake O’Hara, Canadian Rockies^21^, relict Paris Moraine, eastern Canada^22^).

**Figure 3:**
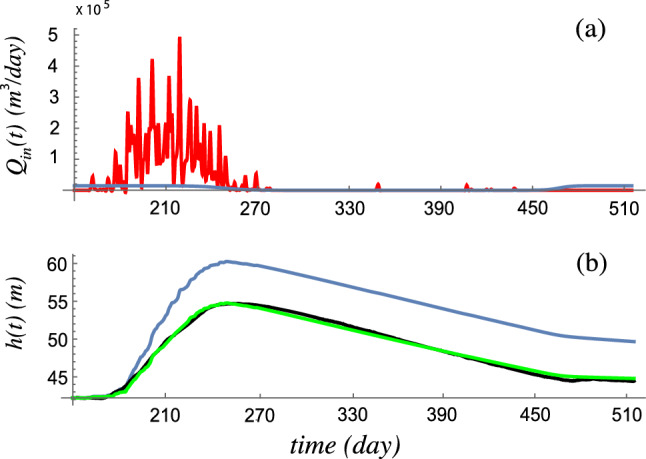
(**a**) Volumetric fluxes (m$^3$/day): from precipitation (red) using the Pyramid Meteo station time-history data from May 31, 2018 to May 31, 2019 and from modeled glacial melt (blue). (**b**) Solutions of Eq. (1), with $K=3.8 \times 10^{-6}$ s$^{-1}$. Curves of water depth vs. time: black—depth data from bottom pressure sensor, blue—model result with full precipitation data, green—model result with 0.63 fraction of precipitation data to match the maximum lake level at day 251 (8 Sept 2018).

Having set $K_h$ , Eq. (1) can be integrated numerically to obtain the water level history *h*(*t*), which in turn can be validated on the data collected by the pressure sensors over the June 2018 through September 2019 time window. Typical results are shown in Fig. 3, where we also depict the influx from the Pyramid precipitation data (which were only available up to June 2019) and the influx from streams fed mostly by glacial melt. Also shown in Fig. 3 are the measured depths, showing good quantitative agreement with the model. It should be stressed that the Pyramid station is located 12 km away from Lake 4, and hence the precipitation rate cannot be expected to be as accurate as if it were collected in a station within the catchment basin of the lake. Consequently, to obtain quantitative agreement we rescale the Pyramid influx data accordingly so that the model matches the measured max water level from our data. We remark that the model may be also run with a different precipitation data set, ERA5. Please see supplementary Fig. S2 for such study. We emphasize that the ERA5 hindcast captures the gross variations in precipitation but differs from the in-situ Pyramid data in overall quantitative lake level variations. See the Supplementary Material section for further discussion.

## Discussion

**Figure 4:**
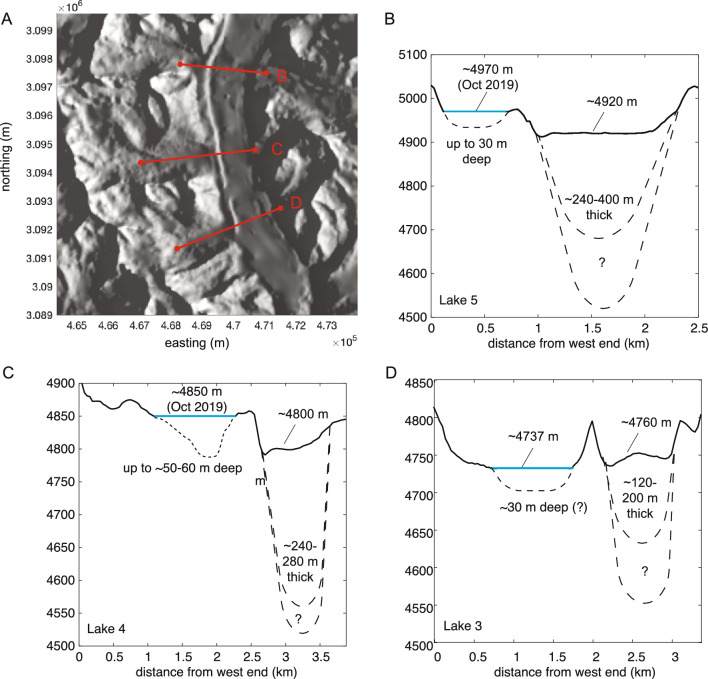
Topography and lake elevations relative to glacier elevations. (**A**) Regional topography, and profile across lake valley and Ngozumpa Glacier for (**B**) Lake 5, (**C**) Lake 4, and (**D**) Lake 3. Note that Lake 5 is perched above the glacier surface, whereas Lake 4 is adjacent to the glacier surface and Lake 3 lies below the glacier surface. Topography data is from NASA SRTM and glacier thickness estimates are from airborne sampling reported in^23^. Lake 3 elevation data is averaged from^16,24^. Lake bathymetry profiles in (**B,D**) are idealized.

Our in-situ data and modeled depth for Lake 4 provide an explanation for the overall behavior of the Gokyo lake system. In particular, lakes 4 and 5 exhibit large-magnitude water-level seasonal variation in drastic contrast to lakes 2 and 3, which remain roughly constant depth throughout the year. Potentially the most significant difference lies in the relative elevation of the lakes with respect to the Ngozumba Glacier. Lake 5 is perched above the surface of the glacier. The surface of Lake 4 lies above the nearby glacier surface, but the lake bed lies at or below that surface (Fig. 4). Both the surface and bed of Lake 3 lie below the surface of the glacier. Further, lakes 4 and 5 also lack surface outflows, which allows them to fill during the monsoons while lakes 2 and 3 continually discharge water through their outflow streams. But following the monsoon, lakes 4 and 5 are likely able to drain more effectively than lakes 2 and 3 due to a relatively lower water table, inferred based on the relative elevation of the nearby glacier (compare Fig. 4B–D), as well as demonstrated through our quantitatively successful hydraulic model. In contrast, the water table likely remains high surrounding Lake 3. Future tracer or subsurface studies (e.g. electroresistivity) would aid in better understanding subsurface and surface flow pathways and rates.

Following the autumn monsoon, Lake 4 cooled during the month of October (Fig. 2G). In November, the lake became well-mixed and continued to cool. Ice formed in mid-December, and the upper layers of the lake became slightly cooler, but remained at the surface because lake waters were all below the temperature of maximum density ($\sim$4 $^\circ$C). During iced-over conditions lake temperatures were approximately 3.5 $^\circ$C. The limited cooling prior to iced-over conditions, and nearly uniform temperatures in the lower portion of the lake (our measurements did not extend to the surface), are consistent with a recently-proposed classification of a ‘cryostratified’ lake^19^. Yang et al.^19^ found that the depth-averaged lake temperature at the time of ice-on ($T_{on}$) is related to $H_{max} A^{1/2}$, where $H_{max}$ is maximum water depth and *A* is the area of the lake. A small lake surface area and large depth allow for little cooling before ice-on, whereas lakes with a large surface area relative to their maximum depth can experience much greater cooling prior to ice-on. These relatively shallower lakes are classified as ‘cryomictic’. Wang et al.^4^ describe seasonal variations of temperature in Nam Co, a large (2020 km$^2$) lake on the Tibetan Plateau at similar altitude to Lake 4, finding minimum temperatures close to 0 $^\circ$C. Yang et al. fit to the relationship between $T_{on}$ and $H_{max} A^{1/2}$ well represents conditions in Lake 4 of 3.62 $^\circ$C at ice-on.

Our analyses show that the water volume change for the 12 meter depth drop we observed in Lake 4 corresponds to a volume of $5.6\times 10^6$ m$^3$. To put this in perspective with reported GLOF (Glacial Lake Outburst Flood) events^10^, volumes in glacial lake outburst floods range over $2\times 10^5$ to $10^7$ m$^3$ of water, though of course the water discharge from Lake 4 happens much more slowly than a GLOF. While case studies of GLOFs have been a focus of other studies and reviews^10^, more seasonal water-level changes are not well/widely documented yet. New technology available (e.g. NASA SWOT Mission) may help with future studies. Studies of glacial melt driven lake dynamics in such high altitude and low latitudes are rare, and likely quite different from their counterpart in polar regions on account of the higher solar forcing, albedo feedbacks, and other effects. Nonetheless, the volume of water stored in the Earth’s ‘third polar’ region clearly merits further careful study due to rapid regional warming trends, and such a detailed analysis provides a benchmark on which to validate remote sensing, and may indicate future potential change due to a warming climate elsewhere on our planet.

## Conclusion

We have performed a systematic investigation of the hydrologic and thermal dynamics of the Gokyo lakes. We find strong differences between those lakes possessing surface outflow (lakes 2 and 3) and those only possessing subsurface outflow (lakes 4 and 5). Lakes 4 and 5 experience dramatic seasonal water level fluctuation, whereas lakes 2 and 3 do not. In fact, our analyses show that the water volume change for the 12 meter depth change we observed in Lake 4 corresponds to a volume of $5.6\times 10^6$ m$^3$, as well as thermal evolution consistent with cryostratified lakes. We conjecture that the relative lake level elevations with respect to the adjacent Ngozumba glacier further control this strong difference in behavior due to those higher lakes being above the water table associated with the nearby glacier. Mathematical modeling applied to Lake 4 provides a predictive means to assess the future impacts of climate change through increased/decreased sources of inflow. Such forecasts are of natural interest both for this particular valley, as well as providing test cases for other similar cryospheric systems poised to experience future climate change. Recent studies have used satellite imagery to document rapid increase in numbers of lakes and their areas, especially supra- and peri-glacial lakes^5^. Our study provides a detailed time history and mechanistic explanation for large variations in lake volumes in one case study.

Having in-situ observations and continuous direct measurement of lake level further has allowed development of our hydraulic model. Level changes of this magnitude probably occur elsewhere. While case studies of GLOFs have been a focus^10^ more seasonal water-level changes are not well/widely documented yet. New technology available (NASA SWOT Mission) may help with future studies. It would be interesting to explore other high mountain lake water level fluctuations driven by seasonal monsoons or longer term climatological factors using selective in-situ observations. We hope that continued mathematical modeling can help assess the likelihood of lake overflow flooding, but additional study including improved photogrammetric mapping may be needed to properly quantify future forecasts. Of course studies of glacial melt driven lake dynamics in such high altitude and low latitudes are rare, and likely quite different from the poles on account of the higher solar forcing, albedo feedbacks, and other effects. Nonetheless, the volume of water stored in the Earth’s ‘third polar’ region clearly merits further careful study on account of rapid regional warming trends.

### Supplementary Information

Supplementary Video 1.

Supplementary Information.

## Data Availability

Raw pressure and temperature time series are available at https://doi.org/10.5281/zenodo.7732886 Lake bathymetry and profile data are available upon request.
