# Supplementary material for: Extreme seasonal water-level changes and hydraulic modeling of deep, high-altitude, glacial-carved, Himalayan lakes
Source: Sci Rep. 2023 Jul 20;13:11705. doi: 10.1038/s41598-023-37667-z (PMC10359347; doi:10.1038/s41598-023-37667-z)
Supplement: Supplementary file 2 — Supplementary Information. [file 41598_2023_37667_MOESM2_ESM.pdf]

Supplementary Materials for
Extreme seasonal water-level changes and hydraulic modeling of deep, high-altitude, glacial-carved, Himalayan lakes

R. Camassa[1*], E.F. Eidam[2*], L.G. Leve[3],
R. M. McLaughlin[1*], H.E. Seim[4*], S. Sharma[5]

[1]Department of Mathematics, University of North Carolina
[2]College of Earth, Ocean, and Atmospheric Sciences, Oregon State University
[3]Department of Religious Studies, University of North Carolina
[4]Department of Earth, Marine, and Environmental Sciences, University of North Carolina,
[5]Department of Environmental Science and Engineering, Kathmandu University

July 18, 2023

This PDF file includes:

Supplementary Text

Fig. S1,S2,S3,S4

Supplementary Text

Here, we present the theoretical details of the hydraulic model for predicting the evolution of the depth of Lake 4 presented in the main body and discussion of the cultural and geographical aspects of the region.

Given the morphology of the terrain where the land-locked Lake 4 is located, it is reasonable to assume that most of the underground outflow from the lake is directed towards the Ngozumba glacier, and is concentrated along the east side of the lake, where the glacier's moraine effectively acts as a dam wall. From our echo sounder data, we construct the curve of this wall's

length vs. depth $L_w(z)$, as well as the hypsometric curve $A(z)$ for the lake, where the origin of the vertical axis z is set at the maximum depth point of the lake, see Figure S1.

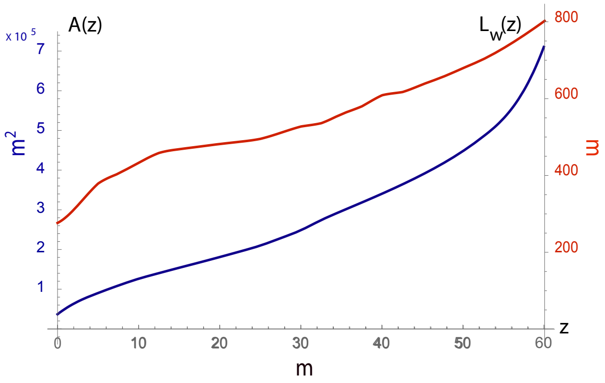

Figure S1: Hypsometric curve $A(z)$ (blue) and East wall perimeter curve $L_w(z)$ (red) vs. height

For a model of the overall water balance in Lake 4, we assume the underground discharge volumetric flux $q_{\text{out}}(z,t)$ at time t is governed by the hydrostatic pressure at the height z and takes place through the infinitesimal area element $L_w(z)dz$ of the east wall. Thus, by Darcy's law and by assuming a constant hydraulic conductivity K_H, the volumetric flux through this area element is

$$q_{out}(z,t) = K\big(h(t)-z\big)L_w(z)dz\,, \tag{1}$$

where we have defined the proportionality constant $K \equiv K_H/L_g$, with L_g the (average) distance of the east wall from the glacier, and $h(t)$ is the lake's surface location at time t so that $h(t)-z$ is the height of the water column above z at that time. The total volumetric outflow $Q_{out}(z,t)$ flux is therefore given by the integral

$$Q_{out}(t) = \int_0^{h(t)} q_{out}(z,t)dz = K\int_0^{h(t)} \big(h(t)-z\big)L_w(z)dz = K\int_0^{h(t)} A_w(z)dz$$

where the last equality follows from an integration by parts and the definition of the underwater lateral area on the moraine side $A_w(z)$ at height z

$$A_w(z) = \int_0^z L_w(s)ds\,.$$

The total influx from the catchment basin precipitation plus hanging glaciers' melt (both surface streams and groundwater) is in principle obtainable by existing data through time-history of weather stations and local measurements. Let this influx be denoted by $Q_{in}(t)$. Then the time evolution of the lake level $h(t)$ is governed by the conservation law

$$\frac{dV}{dt} = A(h)\frac{dh}{dt} = Q_{in} - Q_{out} = Q_{in}(t) - K\int_0^{h(t)} A_w(z)dz\,,$$

where $V(t)$ is the total volume of water in the lake at time t and $A(h)$, the area of the surface of the lake at height h, is given by the hypsometric curve; this yields the model differential equation

$$\frac{dh}{dt} = \frac{1}{A\big(h(t)\big)} \left(Q_{in}(t) - K \int_0^{h(t)} A_w(z)dz \right) . \tag{2}$$

While in principle the integral can be eliminated by taking an overall time derivative of this expression, the resulting second order nonlinear equation does not lend itself easily to analytical approaches, and for a numerical solution it is more convenient to treat the last term in equation (2) as a given function of height $V_w(h)$ obtained by computing the double integral

$$V_w(h) \equiv \int_0^h A_w(z)dz = \int_0^h \int_0^z L_w(s)ds\, dz$$

from our lateral-wall length data $L_w(z)$.

During the 2018 pre-monsoon dry season, from June 6 through June 26, our data show that the water level was approximately constant, i.e., $\dot{h} = 0$, at the (equilibrium) height $h \equiv h_e \simeq 45$ m. The portion of incoming flux, Q_e say, during this time must have then compensated the outflow, and equation (2) can thus be used to solve for K in terms of Q_e at this equilibrium height,

$$K = Q_e \Big/ \int_0^{h_e} A_w(z)dz = \frac{Q_e}{V_w(h_e)} . \tag{3}$$

Similarly, our data show an essentially constant rate of decrease $\dot{h} \equiv -\sigma$ of the water level in the winter months of 2019, when the influx from precipitation and glacial melt can be expected to be negligible.

Setting $Q_{in}(t) = 0$ for t in the time interval corresponding to the low temperatures, as indicated by our lake thermistor data, and combining equations (2) and (3) would therefore require

$$\frac{\sigma}{Q_e} = \text{const.} = \frac{1}{A\big(h(t)\big)} \int_0^{h(t)} A_w(z)dz \Big/ \int_0^{h_e} A_w(z)dz = \frac{1}{V_w(h_e)} \frac{V_w\big(h(t)\big)}{A\big(h(t)\big)} \tag{4}$$

The variation of the right-hand-side (RHS) of this relation with respect to the height h in the range between $h = 44.46$m and $h = 54.68$m, where our data show constant slope, confirms that the RHS varies little around its mean value, with maximal deviations of about 6%. Taking this mean value and computing the slope σ from our data thus determines Q_e and hence K. From our depth data time history between June 2018 and June 2019, these values turn out to be, respectively, $\sigma \simeq 5.36 \times 10^{-7}$ m/s, $Q_e \simeq 0.17$ m^3/s and $K \simeq 3.81 \times 10^{-6}$ s^{-1}. We stress that this is an approximation in that right hand side of equation (4) is not exactly constant but varies from its mean by about 6 percent. Estimating the average distance between the east wall and the glacier as $L_g \simeq 1000$ m yields a value for the hydraulic conductivity $K_H = 0.43 \times 10^{-4}$ m/s, or

$K_H = 37.42$ m/day, which is in line with field-experimental measurements of conductivity for glacial tills (e.g., Eklutna Valley, southcentral Alaska [2], Lake O'Hara, Canadian Rockies [4], relict Paris Moraine, eastern Canada [1]).

We also report the model results on a longer time span using precipitation data by the ERA5 dataset, which is collected with hindcast techniques, in contrast with the in-situ data collected at the Pyramid station. The model results and precipitation data are shown in Figure S2. Note that the spatial scale of ERA5 data set is 9 km by 9 km box, which is coarse and roughly the size of the catchment basin. A few remarks are necessary here. First observe the lack of a constant depth phase at the beginning of the evolution here due to ERA5 reported precipitation which is absent in the Pyramid data. Second, notice that the model evolution with the ERA5 data shows the linear lake decay observed in the mooring data being interrupted with a secondary plateau, and that the minimum lake level predicted by the model with ERA5 precipitation is higher than the one in our data (about 48 m vs. the observed 44.46 m). This is presumably arising from ERA5 data showing excessive pre-monsoon precipitation around day 390 again not present in the Pyramid data. Also noticeable from the model evolution with ERA5 precipitation is the prediction of higher maxima lake levels after the 2019 and (especially) the 2020 monsoon seasons.

With a well tuned mathematical model we may forecast the maximum lake depths assuming a linear extrapolation of the lake hypsometry. The response to an increased monsoon can be quickly assessed in the model by doubling the influx source, etc. Our findings show a sublinear growth of the depth as a function of source inflow, following $h \propto (Q(t)/Q_0)^{0.15}$, for example a doubling of the source yields a net depth increase of approximately 8 m.

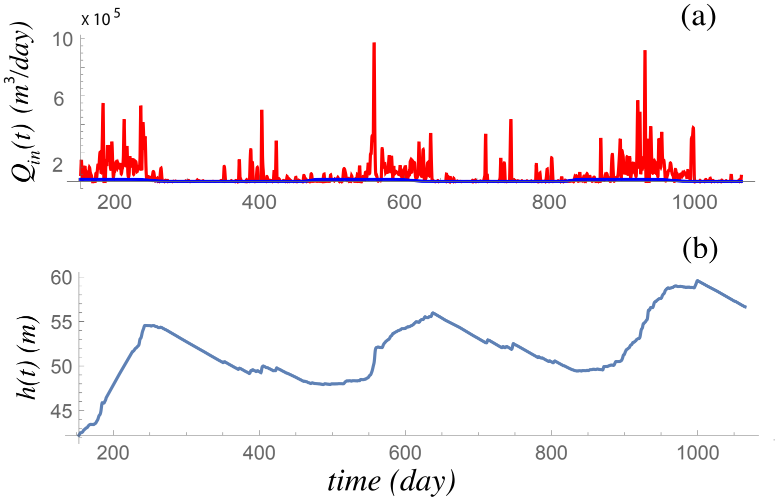

Figure S2: (a) Volumetric fluxes (m^3/day): from precipitation (red) using the ERA5 dataset time-history from May 31, 2018 to Dec. 31, 2020 and from modeled glacial melt (blue). (b) Solution of equation (2), with $Q_e = 0.17$m^3/s and $K = 3.8 \times 10^{-6}$s^{-1} (corresponding to a hydraulic conductivity $K_H = 0.43 \times 10^{-3}$m/s, or $K_H = 37.42$m/day).

We next provided additional description for the methods used to collect in situ data from

Lake 4 and the two precipitation data sets we used.

First, lake measurements were collected in May/June 2018 and October 2019. During both surveys, bathymetry data were collected from several lakes using a Deeper Pro echosounder towed from a 10-ft inflatable boat.

A string of 10 Onset HOBO water temperature Pro v2 (U22-001) data loggers, bounded top and bottom by Onset HOBO water level data loggers (U20L-002 above and U20-001-03 below), spaced approximately 4 m apart, were deployed as a vertical mooring in early June 2018 (27°48'35.8"N, 86°40'55.6"E), see Figure 2 from the main text to monitor water temperature and water level variations in Lake 4. The mooring was suspended under a subsurface float and anchored with a bag of rocks. Each sensor collected observations every 15 minutes. The mooring was recovered and observations downloaded in October 2019.

In October 2019, profiles of conductivity, temperature, depth, turbidity, and uncalibrated chlorophyll-a were measured using an RBR Concerto sensor outfitted with Seapoint optical backscatter sensor (OBS).

Next, the bathymetry of Lake 4 was established by combining soundings measured with the Deeper Pro echosounder with discrete depth measurements from the RBR profiler and satellite-derived topography (NASA SRTM 90-m resolution; earthdata.nasa.gov) of the area surrounding the lake (see Figure S3). A lake perimeter was extracted from Google Earth imagery collected during a time of relatively high water level, to bound the bathymetric interpolation. Hypsometry (area versus elevation) was derived at 2.5 m increments from the lake bottom to surface.

We utilized two distinct precipitation data sets in our modeling effort below. First, observations collected at the Pyramid International Laboratory/Observatory (27°57'33"N, 86°48'47"E [28]) located in the Khumbu valley roughly 12 km east of Lake 4 were used to force the model. This provides in situ precipitation data from January 2018 through June 2019. Second, re-analysis data from ERA5-land (www.ecmwf.int/en/era5-land) were downloaded for a grid cell centered over Lake 4 at hourly intervals for the a longer time period and were used to generate a model forecast of varying lake depths.

Temperature and turbidity profiles collected in mid-October find lakes 2, 4 and 5 to be thermally stratified, with a weakly-stratified surface layer separated from a weakly-stratified bottom layer by a distinct thermocline at roughly mid-depth (Figure S4). The temperature change across the thermocline was approximately 3° C in lakes 4 and 5 and half as large in lake 2. Turbidity was highest in lake 4, with largest values below the thermocline. Turbidity was less in lake 5, also with largest values below the thermocline, whereas turbidity was significantly lower in lake 2 with slightly larger values above the thermocline.

Geographical and Cultural Aspects of the Region: We note that all five lakes lie within the official boundaries of Sagarmatha National Park, in the ancestral territory of the ethnic Sherpa Buddhist community. According to Sherpa Buddhism, the mountain landscape is home to a diverse variety of supernatural agents who reside in unique features of the natural environment such as mountain peaks, rivers and lakes [3, 6, 5]. These creatures are believed to help humans when they are pleased but to cause massive destruction when they are disturbed, unleashing

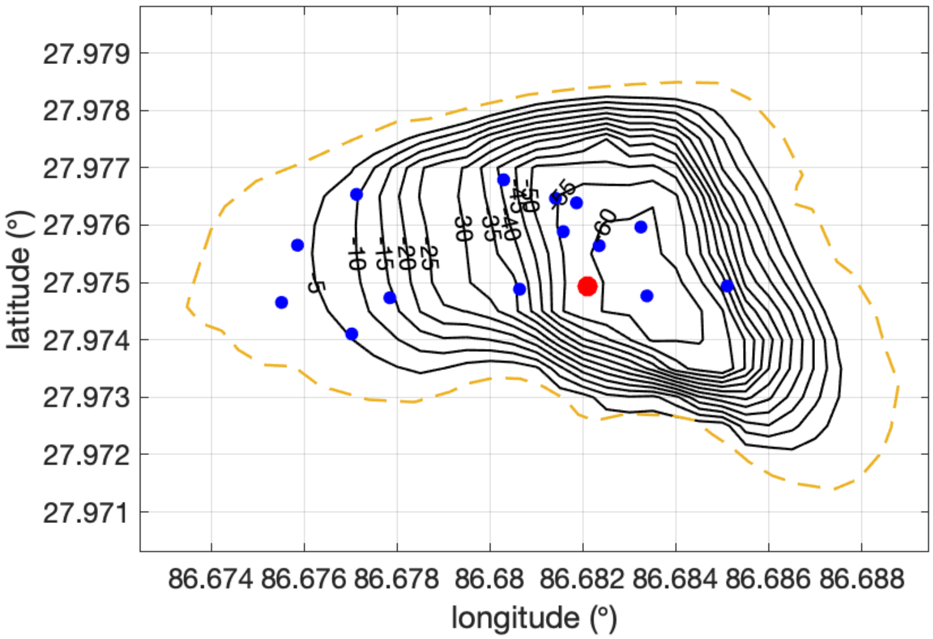

Figure S3: Interpolated bathymetry(in meters) derived from 2019 field measurements. Red circle denotes mooring location (June 2018 to October 2019) and blue circles denote locations of CTD/turbidity profiles measured in October 2019. The dashed outline illustrates a typical shoreline position.

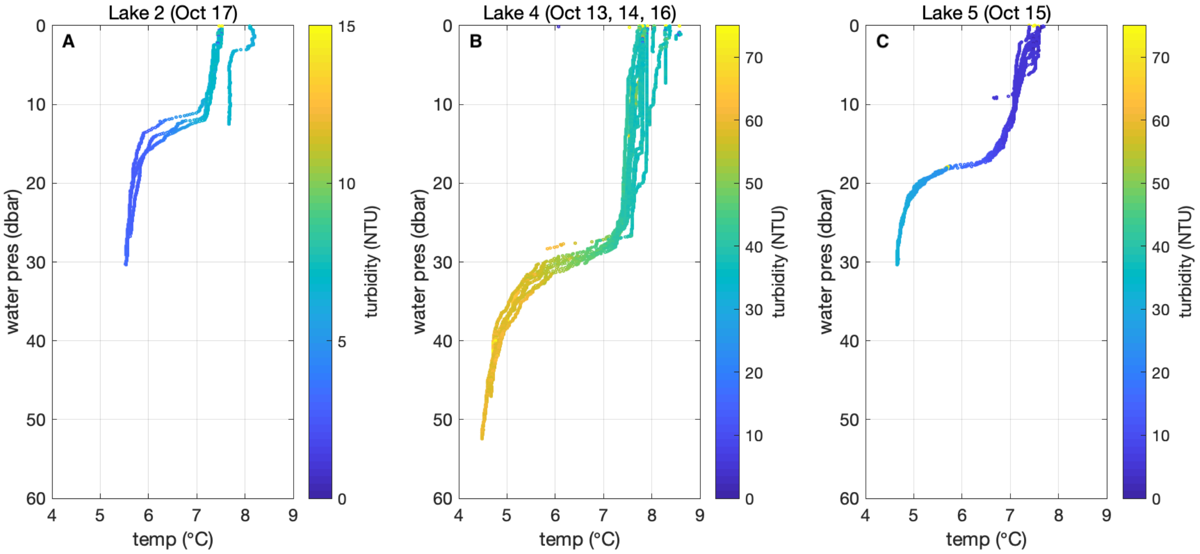

Figure S4: Profiles of lake structure post-monsoon. A) Lake 2, characterized by a $\simeq$1.2° temperature gradient and slightly turbid water in the upper layer. B) Lake 4, characterized by a $\simeq$3° temperature gradient and up to 60 NTU turbidity in the cold, lower layer. C) Lake 5, characterized by a $\simeq$3° temperature gradient and slightly more turbid water at depth. Each plot represents all profiles collected at a given lake during the days noted, at multiple locations. Note that the turbidity scales are different.

floods, landslides and other dangers on already vulnerable communities.

Local tradition holds that all five of the Gokyo lakes are inhabited by such creatures, who are considered sacred and said to be angered by human incursions into the water. In particular, Lake 3 (elevation 4730 m) is thought to house an especially powerful being, on whose goodwill the safety of the adjacent human settlement is seen to depend. For this reason, humans do not fish, swim, boat or bathe in the lake; nor do they break the surface of the water with their hands or feet. Springs which feed the lake are used as the water source for the community via a surficial piped network. However, the lake shore is treated as a boundary between the pure domain of the sacred creature and the profane world of ordinary human activity. Given the importance of these religious and cultural beliefs to the local population, our team did not undertake any scientific exploration on Lake 3 that would have put us in conflict with our host community. Instead, we showed respect for local tradition by focusing data collection activities that required entering the water to Lake 4, with the approval of Sherpa community. It goes without saying that care must be taken in performing scientific measurements to respect local culture.

References

[1] Arnaud, E., McGill, M., Trapp, A., & Smith, J. E. (2018). Subsurface heterogeneity in the geological and hydraulic properties of the hummocky Paris Moraine, Guelph, Ontario. Canadian Journal of Earth Sciences, 55(7), 768-785.

[2] King, A.L., Meyers, J.S. and Brown, J.M. (2019). Hydraulic conductivity of quaternary surficial units within the Eklutna Valley, southcentral Alaska. Journal of Hydrology, 575, 166-174

[3] Klatzel, Frances (2010). Gaiety of Spirit: The Sherpas of Everest. Victoria (BC): Rocky Mountain Books.

[4] Langston, G., Hayashi, M., and Roy, J. W. (2013). Quantifying groundwater-surface water interactions in a proglacial moraine using heat and solute tracers. Water Resources Research, 49(9), 5411-5426.

[5] Ortner, Sherry B. (1978). Sherpas through their rituals. Cambridge ; New York : Cambridge University Press

[6] von Fürer-Haimendorf, Christoph (1964). The Sherpas of Nepal : Buddhist highlanders London : John Murray.
